# Supplementary material for: Molecular hydrogen in the N-doped LuH3 system as a possible path to superconductivity
Source: Nat Commun. 2024 Aug 23;15:7283. doi: 10.1038/s41467-024-51348-z (PMC11343858; doi:10.1038/s41467-024-51348-z)
Supplement: Supplementary file 1 — Supplementary Information [file 41467_2024_51348_MOESM1_ESM.pdf]

Supplementary Information:

"Evidence of Molecular Hydrogen in the N-doped LuH_3 System: a Possible Path to Superconductivity?"

Cesare Tresca,[1,*] Pietro Maria Forcella,[2] Andrea Angeletti,[3,4] Luigi Ranalli,[3,4] Cesare Franchini,[4,5] Michele Reticcioli,[4,†] and Gianni Profeta[1,2]

[1] *CNR-SPIN c/o Dipartimento di Scienze Fisiche e Chimiche, Università degli Studi dell'Aquila, Via Vetoio 10, I-67100 L'Aquila, Italy*

[2] *Dipartimento di Scienze Fisiche e Chimiche, Università degli Studi dell'Aquila, Via Vetoio 10, I-67100 L'Aquila, Italy*

[3] *University of Vienna, Vienna Doctoral School in Physics, Boltzmanngasse 5, 1090 Vienna, Austria*

[4] *University of Vienna, Faculty of Physics and Center for Computational Materials Science, Kolingasse 14-16, 1090 Vienna, Austria*

[5] *Dipartimento di Fisica e Astronomia, Università di Bologna, Viale Berti Pichat 6/2, I-40127 Bologna, Italy.*

Contents

Supplementary Note 1. Ramping-temperature MLFF-MD calculations 4

Supplementary Note 2. Additional MLFF-MD Data 6

Supplementary Note 3. Bader Charge Analysis 9

Supplementary Note 4. MLFF-MD on the Undoped Compound 12

Supplementary Note 5. Mapping to the representative $2 \times 2 \times 2$ sub-system 14

A. More on the accuracy of the model adopted 14

Supplementary Note 6. Electronic correlations treatment 16

Supplementary Note 7. More on the dynamical properties 19

Supplementary Note 8. Calculation of the superconducting critical temperature 24

Supplementary Note 9. Accuracy of MLFF-MD calculations 25

Supplementary Note 10. Comparison with XRD and Raman experimental spectra 26

Supplementary References 28

List of Figures

1 Ramping-T MLFF-MD simulations 4

2 MLFF-MD representative structures 5

3 Pair correlation functions at 100, 200 and 300 K in panel a, b, c, respectively, for H-H, Lu-H, Lu-Lu pairs. 6

4 Trajectory of hydrogen atoms at 300 K 7

5 H-H bond length in MLFF-MD 9

6 Number of H_2 molecules in MLFF-MD at 100, 200 and 300 K 10

* cesare.tresca@spin.cnr.it

† michele.reticcioli@univie.ac.at

7 Density of States of MD structures . . . 11
8 MLFF-MD on the Undoped Compound . . . 13
9 Electronic Band Structure . . . 15
10 Electronic dispersions and density of states (left) and dynamical properties and Eliashberg Function (right) of the $LuH_{2.875}N_{0.125}$ with two molecules structural relaxed up to theoretical P^{DFT}=0 kbar pressure. . . . 16
11 Hubbard correction to the density of states . . . 18
12 Hubbard term for the Lu d-states . . . 19
13 Density of states with and without U . . . 20
14 Phonon Properties . . . 21
15 Electronic response to selected phonon modes . . . 22
16 We report the Bader charge analysis as obtained from VASP calculations as a function of the atomic displacements along the phonon eigenvectors we are interested in. The average value of the Bader charge is reported for every element (in units of the electronic charge, e). . . . 23
17 SCDFT . . . 24
18 MLFF-MD accuracy. . . . 25
19 XRD: Theory vs experiment . . . 26
20 Raman: Theory vs experiment . . . 27

List of Tables

I Bader Charge Analysis . . . 12
II Influence of the U parameters to the Bader charge analysis . . . 19

Supplementary Note 1. Ramping-temperature MLFF-MD calculations

Fig. 1 shows the results obtained from our machine-learning-accelerated molecular-dynamics calculations, with a temperature ramping from ∼0 K to 400 K ($50{\cdot}10^3$ steps). A portion of these data is shown in Fig. 1 in the main text (initial 200 fs), to highlight the formation of H_2 molecules at low temperature. As discussed in the main text, we started the simulation from a highly symmetric Fm$\bar{3}$m $LuH_{2.875}N_{0.125}$ $(4 \times 4 \times 4)$ unit cell (shown in Fig. 2a); H_2 molecules start to form after a few tens of fs, at temperatures as low as 15 K (see also Fig. 1 in the main text). Fig. 2b shows the structure with the H_2 molecules, as obtained from this MLFF-MD calculation. The steep temperature increase observed during the initial stage of the ramping process (at time $t << 1$ ps in Fig. 1c) is attributed to the significant reduction in potential energy within the system (Fig. 1b), due to the rapid formation of H_2 molecules.

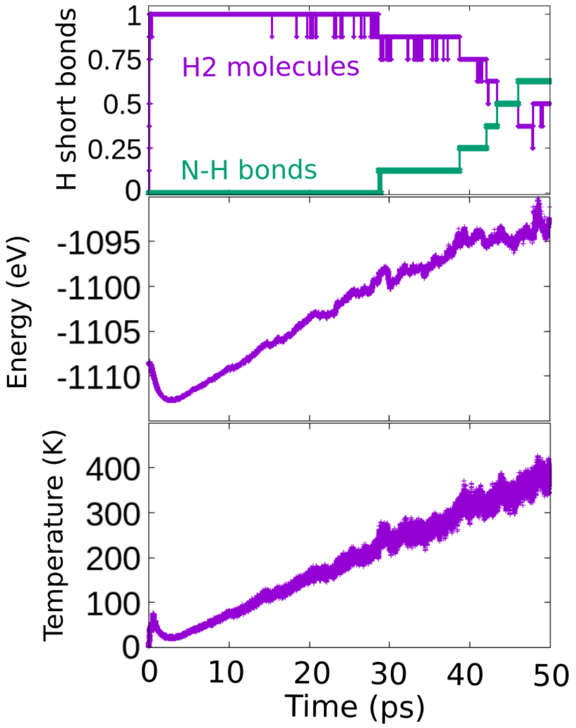

Supplementary Figure 1. MLFF-MD simulations with temperature ramping from 0 K to 400 K. Number of H_2 molecules (purple) and N-H bonds (green) per N atom in the unit cell (top panel), free energy (central panel) and effective temperature (bottom panel). The formation of H_2 molecules in the initial 200 fs are shown in the Fig. 1 in the main text.

Once the molecules are formed and stabilized, the temperature rises following the expected linear ramping. At high temperature (approximately 250 K) the H_2 molecules start to dissociate (see Fig. 1a at time steps $t > 25$ ps). As discussed in the main text, this process is associated to the formation of H-N bonds: Fig. 1a shows the progressively increasing number of H-N bonds at high temperature (a representative structure is shown in Fig. 2c).

The hydrogen atoms move away from the high symmetry positions they occupy in the ideal Fm$\bar{3}$m structure, leading to a symmetry reduction. The structures containing H_2 molecules (one or two) are intrinsically disordered, without any high structural symmetry, thus the space group is P1. See the appendix for the structures of the reported systems.

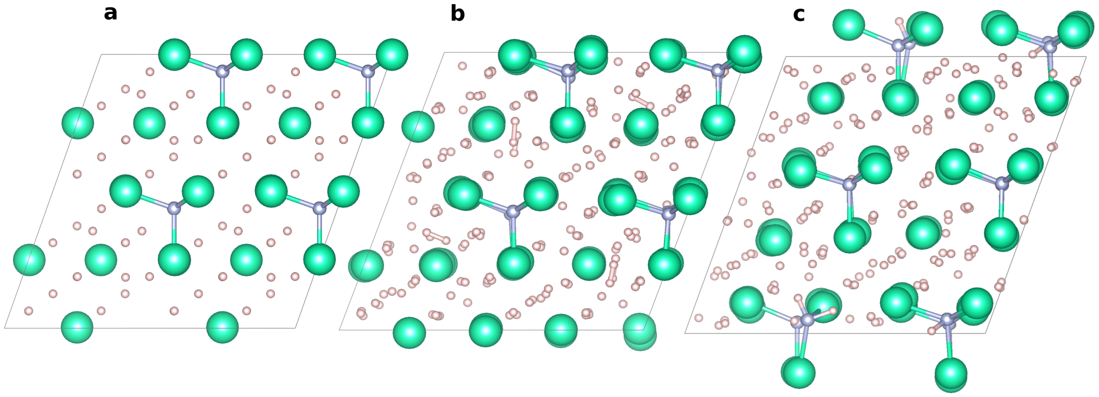

Supplementary Figure 2. MLFF-MD representative structures from the simulation with ramping temperature. We show the initial structure with the crystal in the Fm$\bar{3}$m phase (panel (a)), the structure after the formation of H_2 molecules at low temperature, T$\sim$ 50 K (panel (b)), and the structure showing the presence of N-H and N-H_2 bonds at high temperature, T$\sim$ 400 K (panel (c)).

Supplementary Note 2. Additional MLFF-MD Data

In this section we present additional data on N-doped LuH_3 as obtained from our MLFF-MD simulations.

Figure 3 shows the pair correlation functions as obtained for the molecular dynamics simulations at 100, 200 and 300 K. The sharp peaks indicate strong correlations at specific distances that match the material's crystal lattice parameters (as well as the H_2 bond length for molecular hydrogen). These peaks diminish in intensity with increasing distance, especially for the disordered H atoms (sitting on/around lattice sites or forming molecules), but remain visible up to large distance. This analysis substantiates the stability of the proposed structure.

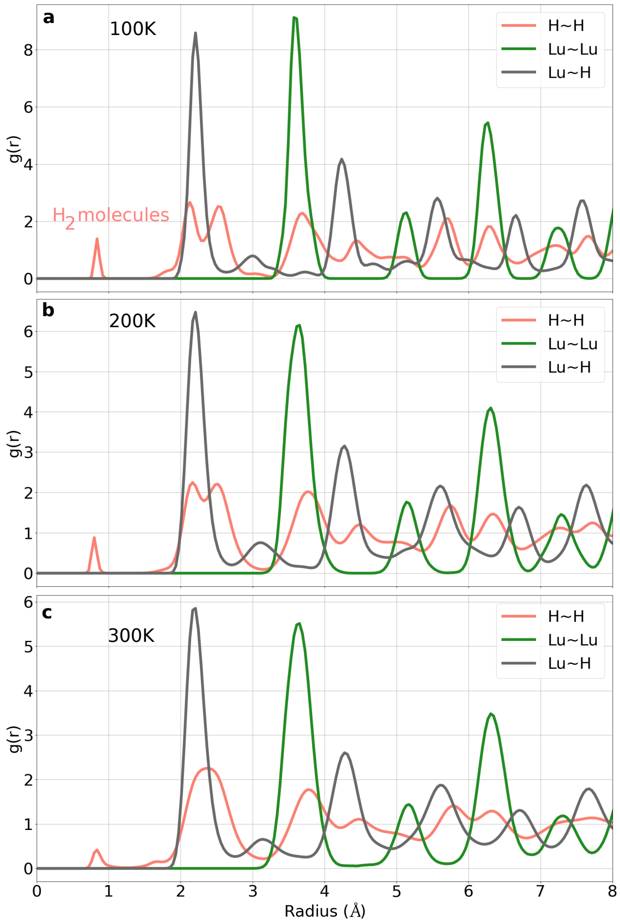

Supplementary Figure 3. Pair correlation functions at 100, 200 and 300 K in panel a, b, c, respectively, for H-H, Lu-H, Lu-Lu pairs.

We note also that, upon formation of H_2 molecular units, some of the H atoms involved in the process present a temporary diffusive behavior. Figure 4 shows the time evolution of selected hydrogen atoms at 300 K. Atoms not involved in the molecule formation vibrate around their equilibrium site (see atom H^0); conversely, some of the atoms forming molecules move away their original position during the formation process as well as upon breaking of the molecular bonds. This might possibly suggests an increased mobility that can contribute to a ionic conductivity: It might be interesting in future studies to explore the impact of such ionic conductivity on the electronic properties of the system, for example in relationship to the resistivity drop recently observed in the literature [1]. We observe similar behavior also in the simulations at lower temperatures, upon formation and breaking of molecular bonds.

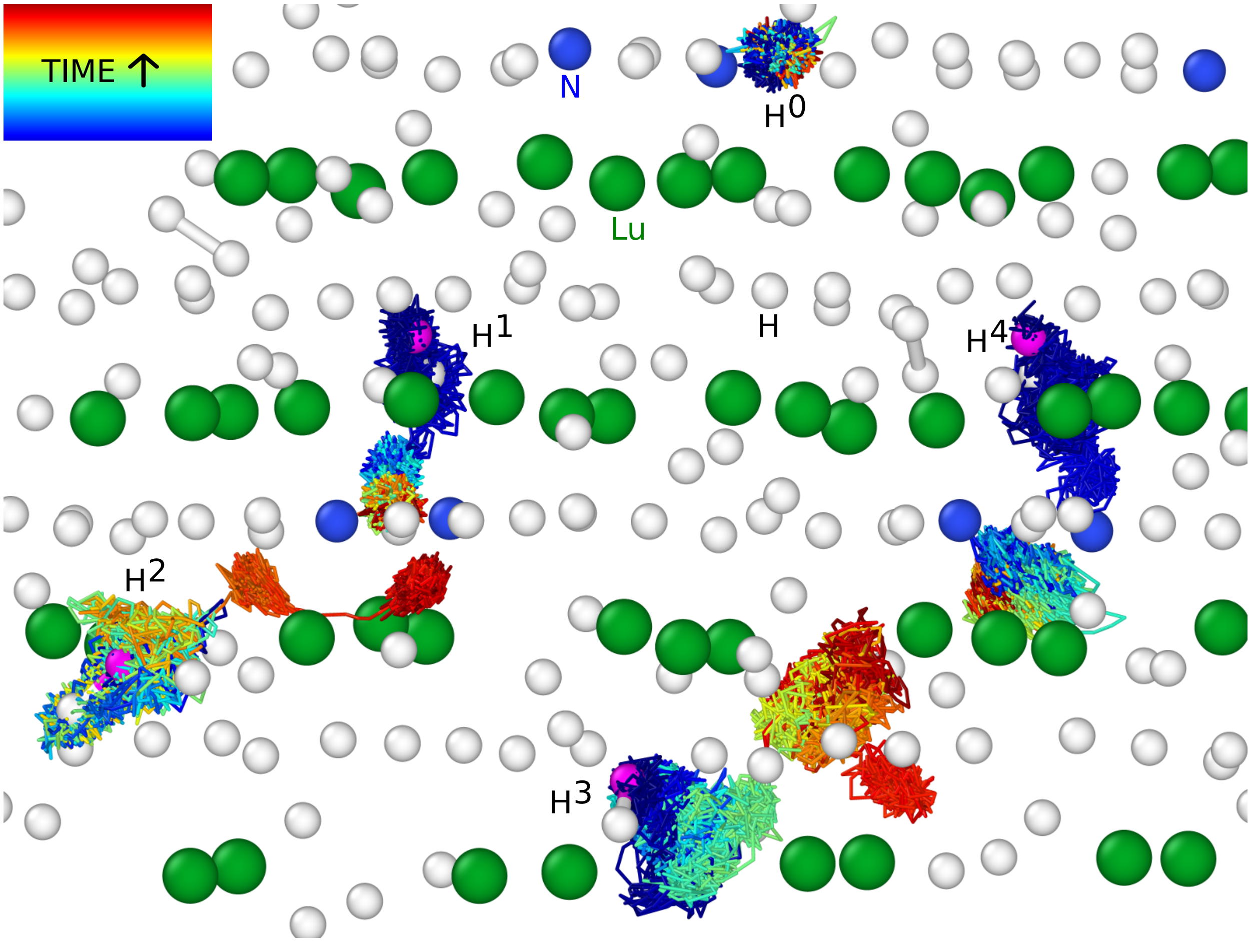

Supplementary Figure 4. Trajectory of selected hydrogen atoms highlighted in fuchsia (in their initial position), at every 10 fs interval, during 0.3 ns long simulation at 300 K. The atom H^0 representative of the almost totality of the hydrogen atoms, only oscillates around its fixed sites. H atoms involved in molecular bonds may sporadically diffuse away from their stationary site (H^1–H^4). The gradient color of the trajectories indicates the time direction, blue for initial positions and red for the final ones.

However, these results, are highly affected by the Langevin thermostat and friction coefficient adopted in the NpT calculations. We also note that the structural integrity of the Lu-hosting matrix remains unaffected by any potential hydrogen disorder, maintaining an ordered face-centered cubic (fcc) lattice.

Figure 5 a) shows the distance between two H atoms forming a H_2 molecule as a function of time (in the MLFF-MD at 300 K). The average value of the H_2 bond-length is 0.83 Å (see solid line in the figure), approximately 10% larger than the bond-length value of 0.74 Å

obtained for the gas phase by DFT calculations (dashed line).

Figure 5 b) shows the total volume oscillation of the supercell employed in the calculations. Once the temperature has stabilized at the desired value, we have observed a corresponding variation of only 2%, around the average volume value, throughout the entire NpT simulation. In order to achieve this we have employed the Langevin thermostat with a fictitious mass for the lattice degrees-of-freedom of value 1000 amu and a friction coefficient for lattice degrees-of-freedom of 10 ps^{-1}.

Figure 6 shows the number of H_2 molecules per N as function of time in the MLFF-MD simulations at 100 and 200 K. At 100 K all molecules survive for the entire simulation. At 200 K some of the molecules dissociate, reaching a number similar as in the simulation at 300 K (see Figure 1 in the main text).

As discussed in the main text, the number of H_2 molecules and H-N bonds determines the metallic/insulating state of the system at every time step: When the total number of short hydrogen bonds (H_2 and N-H) is different from the total number of nitrogen atom in the unit cell, the density of states shows a metallic character. Figure 7 shows the density of states (and a snapshot of the electronic bands around the Fermi level near Γ) of a representative metallic structure explored in the MLFF-MD simulation (the internal forces were not relaxed, *i.e.*, the atomic structure was kept at the MD positions). We have calculated the density of states for various structures explored in the MLFF-MD simulations, and marked the corresponding metallic regimes in Figure 1b in the main text.

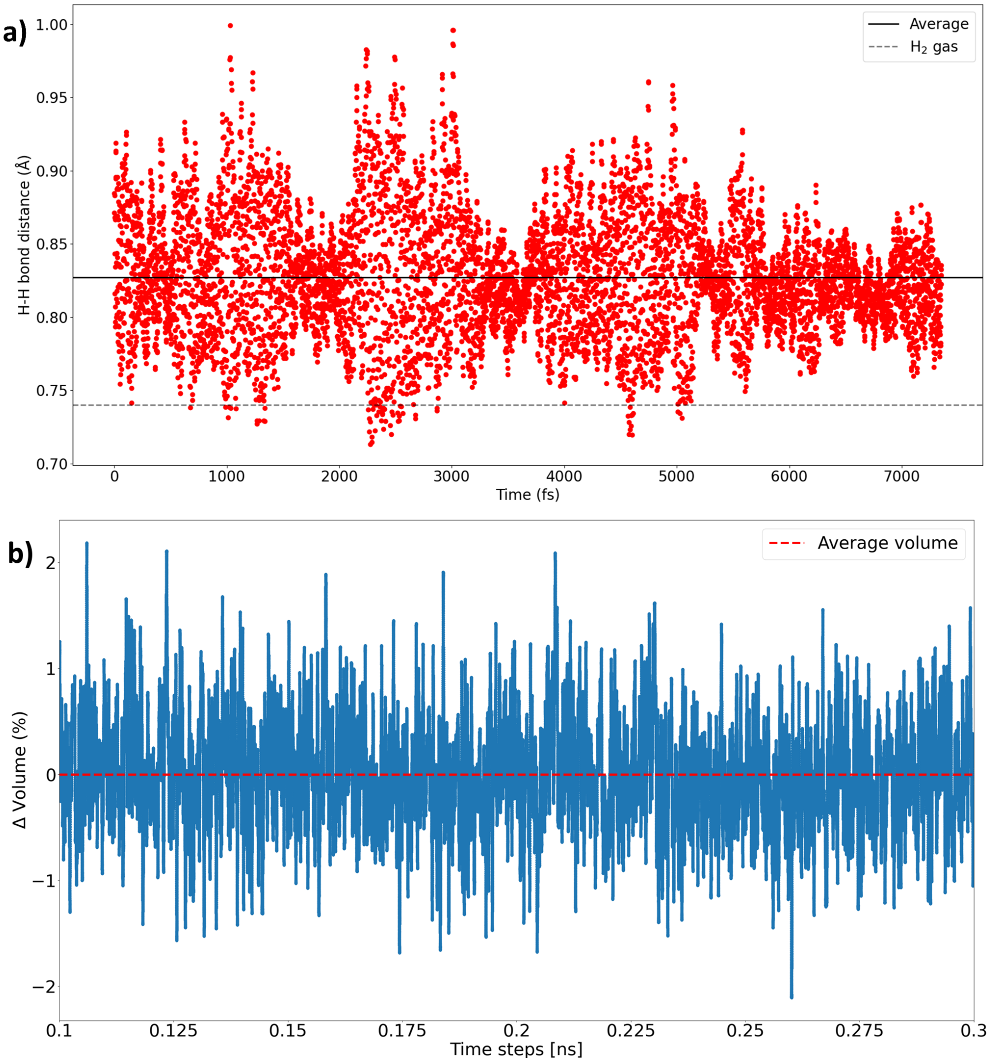

Supplementary Figure 5. a) Distance between two hydrogen atoms in a H_2 molecule as a function of time. Data refer to the MLFF-MD simulation at 300 K. The solid line represents the average bond-length between two hydrogen atoms in a H_2 molecule, while the dashed line represents the bond-length calculated in the gas phase. b) Percentage of volume variations in region of interest of the MLFF-MD during the which transition between the insulating and metallic phase occurs.

Supplementary Note 3. Bader Charge Analysis

Table I shows the Bader charge analysis as obtained from `VASP` calculations for the N-doped LuH_3 system, comparing different cases.

In models including a number of short H-H ($\simeq 0.8$ Å) and N-H ($\simeq 1.0$ Å) bonds equal to the number of N dopants, the system show an insulating behavior. In such systems, we assign

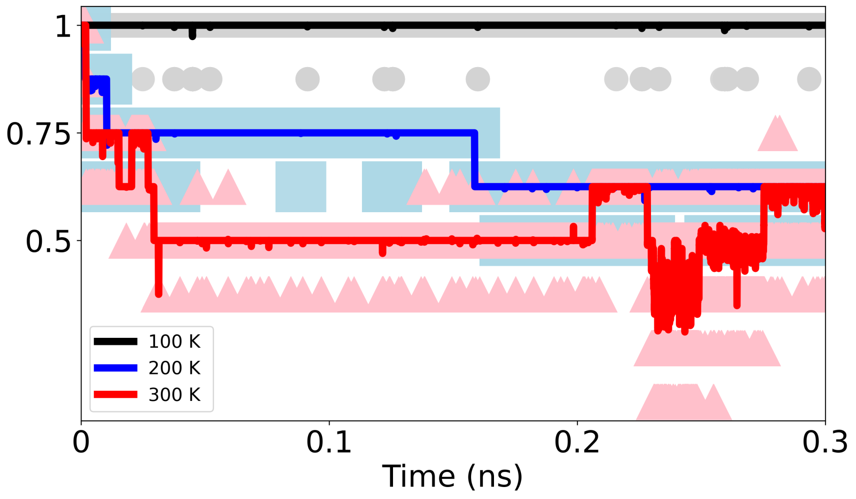

Supplementary Figure 6. Number of H_2 molecules per N atom in the MLFF-MD simulations at 100, 200 and 300 K. We used a threshold distance of 1.0 Å to consider a H-H pair as a H_2 molecule. The circles, squares and triangles indicate the number of H_2 molecules found at every time step in the simulations at 100, 200 and 300 K, respectively. The solid lines represent the running average (calculated over 100 fs).

a valence state of +3 to Lu atoms, and −1 and −3 to H and N atoms not involved in the short bonds, respectively. The N-H bond does not perturb to a sizable extent the N valence state, while the H atom in this configuration tends to donate its electron to the crystal. The molecular hydrogen atoms do not share large amount of charge with the crystal, showing a small electron doping (Bader charge of 1.1 e per H atom in the H_2 molecule) responsible for the elongation of the molecular bond.

In case the number of short H-H and N-H bonds differ from the number of N impurities, then we observe metallic states. In these configurations, the excess electronic charge is accommodated on the Lu orbitals (the Bader charge goes from 7.3 e in the insulating systems to 7.4 e), in perfect agreement with the electronic band structure and density of states shown in the main text. Whether the N atom is substituted on tetragonal or octahedral sites of the Lu lattice seem not to affect the results for the Bader charge.

The Bader charge analysis contributes to clarify the stabilization of the metallic-vs-insulating character of the system upon formation of short H-H and H-N bonds. The 3 electrons donated by the Lu^{+3} atoms are transferred to the H^{-1} atoms in the undoped system. By substituting one H^{-1} atom, the N^{-3} dopant can accommodate two additional

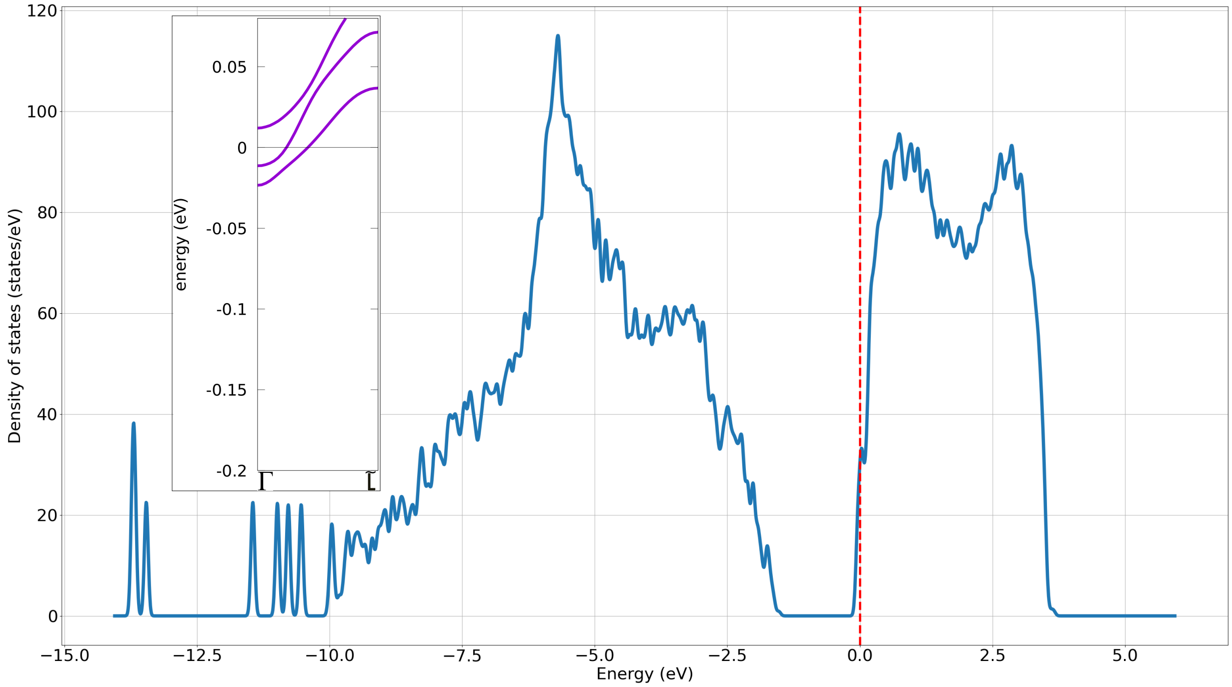

Supplementary Figure 7. Density of states of a representative metallic configuration extracted from the MLFF-MD at time steps showing a number of H_2 molecules and H-N bonds different to the number of N atoms in the unit cell. The internal forces were not relaxed (the atomic structure was kept at the MD positions). The red dashed line indicates the Fermi energy. The inset shows the corresponding band structure in the supercell folded Γ–$\tilde{L}$ path, where $\tilde{L} = (0.5, 0.5, 0.5)$.

electrons as compared to the H atom in the pristine cell: thus, two other H atoms are now not involved in any sizable charge transfer, and can form a molecular-like H_2 complex (with a Bader charge of 1.1 e per H atom, *i.e.*, one electron per H atom as in the gas phase, plus a small $-\varepsilon$ perturbation induced by the crystal). In this situation, the system is insulating. Similarly, H-N short bonds might form instead of the H_2 molecule, keeping the system insulating as the result of the H atom donating its electron to the crystal (the H atom tends to a H^{+1} valence state, showing a Bader charge of 0.6 e). In case the number of short H-H and H-N bonds is not commensurate with the number of N impurities, our systems show a metallic character, with excess electronic charge occupying Lu orbitals ($Lu^{+3-\delta}$).

size	model	el. charac.	Lu	H	H in H_2	H in H-N	N	N in H-N
2×	1 H_2, 1 N tetra	insulating	7.3 $(+3)$	1.6 (-1)	1.1 $(-\varepsilon)$	-	6.6 (-3)	-
2×	2 H_2, 1 N tetra	metallic	7.4 $(+3-\delta)$	1.6 (-1)	1.1 $(-\varepsilon)$	-	6.6 (-3)	-
2×	2 H_2, 1 N octa	metallic	7.4 $(+3-\delta)$	1.6 (-1)	1.1 $(-\varepsilon)$	-	6.6 (-3)	-
4×	3 H_2, 8 N, 5 N-H	insulating	7.3 $(+3)$	1.6 (-1)	1.1 $(-\varepsilon)$	0.6 $(+1-\theta)$	6.6 (-3)	6.6 (-3)

Supplementary Table I. Bader Charge Analysis. Every line refer to a different model, built using either a $2\times2\times2$ (2×) or $4\times4\times4$ (4×) unit cell, including 1, 2 or 3 H_2 molecules, N substitutional doping in tetragonal (tetra) or octahedral (octa) sites, and 5 N-H bonds in the larger cell only (with all N in tetragonal sites). All structures were relaxed to minimize the internal forces (see Methods); the Bader charge and the (metallic or insulating) electronic characters were evaluated from the density of states of the relaxed structures. The average value of the Bader charge is reported for every element (in units of the electronic charge, e), separately; H and N atoms forming short (H_2 and N-H) bonds are distinguished from the rest of the atoms in the crystal. The corresponding valence state value in the ionic bond picture is reported in parenthesis (the reference number of valence electrons of every species in its neutral state reads 9 for Lu, 1 for H and 5 for N); the δ, ε and θ symbols refer to small charge deviation from the ideal values.

Supplementary Note 4. MLFF-MD on the Undoped Compound

In addition to the MLFF-MD simulations described in detail in the main text, we performed further calculations to inspect the role of nitrogen atoms in the stabilization of the H_2 molecules. To this purpose, we performed MLFF-MD calculations of the undoped LuH_3 compound, starting from the system either in the Fm$\bar{3}$m phase or in a structure including an high number of manually created H_2 molecules (16 molecules in the $4\times4\times4$ unit cell, corresponding to 2 molecules per $2\times2\times2$).

Starting from the Fm$\bar{3}$m phase we do not observe formation of any molecules (results not shown), while in Figure 8 we report the results obtained from an initial guess in which molecules were artificially formed. Relaxing the internal forces to the local energy minimum, we observe that the H_2 molecules remain still unbroken. However, during the molecular dynamics simulations all molecules have rapidly dissociated (as shown in the Figure for the

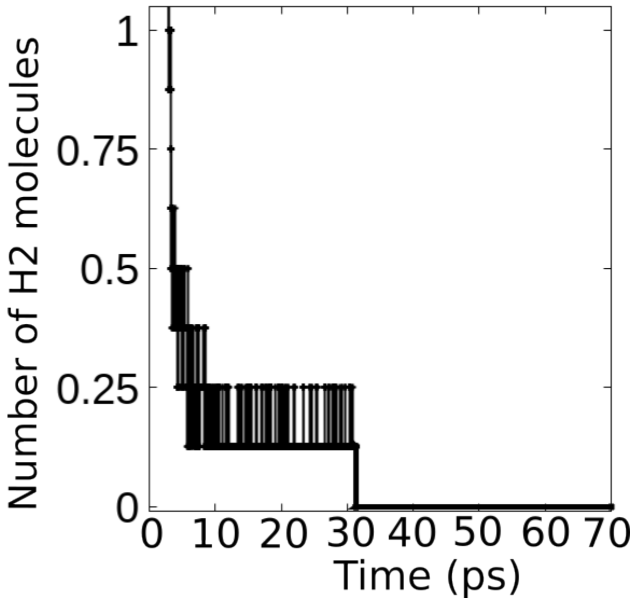

Supplementary Figure 8. Number of H_2 molecules in the undoped LuH_3 $4 \times 4 \times 4$ unit cell in the MLFF-MD simulation at 300 K. For better comparison with the N-doped case, the number of H_2 molecules is reported per $2 \times 2 \times 2$ cell.

simulation at 300 K) and after $\sim$ 30 ps the molecules have completely disappeared.

Supplementary Note 5. Mapping to the representative $2 \times 2 \times 2$ sub-system

To study in more details the effect of H_2 molecules, due to the huge computational cost in study the $4 \times 4 \times 4$ system (including ∼250 atoms), we have modelled two structures representative of the stable MLFF-MD phase, by selecting sub-units in a $2 \times 2 \times 2$ unit cell containing one or two H_2 molecules per N atom and then relaxing the structures in their local energy minimum (see Fig.9 and 2 for comparison).

The phase with one H_2 molecule results lower in energy with respect to the Fm$\bar{3}$m structure (however, it is dynamically unstable as discussed in the next Section). On the other hand, the one containing two H_2 molecules results energetically higher (0.3 eV/Lu) than the Fm$\bar{3}$m structure (because of the smaller unit cell with respect to the one used in the MLFF-MD calculations, which does not allow for a complete description of structural disorder). Consistently with the Bader charge analysis, we have found that the system with one H_2 molecule formed is an insulator, presenting nearly flat bands and van Hove singularities (see Fig.9(bottom-left)) at -2.5 eV. On the other hand, as discussed in the main text, with two H_2 per unit cell the system is metallic and thus explored for possible superconducting properties.

Thus, as described in the main text, the metallic-insulator behaviour is essentially governed by the presence of one (insulator) or two (metallic) molecules per N-atom. This aspect has been substantiated by the band structure calculations for different metastables configurations (not shown), in agreement with the Bader charge analysis (Sec.III of the SM).

A. More on the accuracy of the model adopted

Selecting the experimental lattice constant is the most natural choice to build a representative model for an intrinsically disordered system. Obviously, this leads to a low residual virtual pressure (P$\sim 40 \div 80$ kbar). In order to correctly evaluate the enthalpy of our model representative of the molecular hydrogen phase, we fully relaxed the structure to reach the computational P^{DFT}=0 kbar (which results in slight deformations of the cubic lattice, ending in a triclinic phase, with an average expansion of about 3% of the lattice constants, with respect the experimental one). This slight deformation practically does not affect the

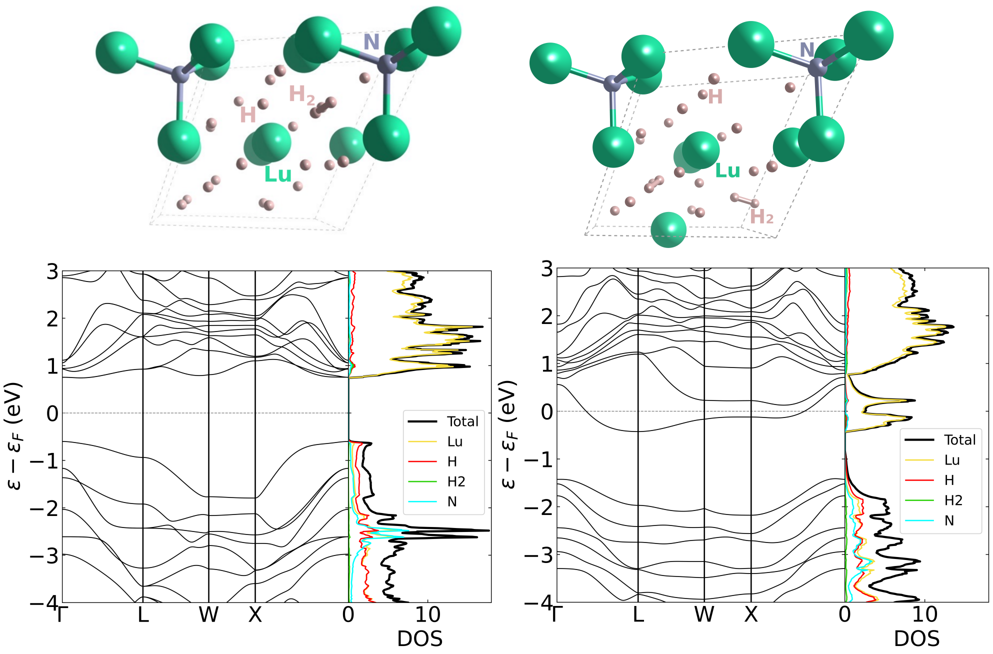

Supplementary Figure 9. In the top panel we report a perspective sketch of both the representative sub-system containing one (left) and two (right) H_2 molecules. In the bottom left panel we report the electronic band structure over the BZ and the projected DOS for $LuH_{2.875}N_{0.125}$ with one H_2 molecule. In the bottom right panel, the same system in a higher-energy configuration involving two H_2 molecules. The atomic contribution to the DOS is highlighted with different colours, see legend.

stability of the molecules nor the electronic properties with respect to the results already reported in the main text for the system at the experimental lattice constant. In particular, the calculated enthalpies of the various models (0, 1 and 2 molecules) considering structural relaxation at zero pressure, are in agreement with the total energies already found for the same models at fixed experimental volume: with respect to the system without H_2 molecules, the formation enthalpy for the system with one H_2 molecule is $\Delta H = -0.010$ eV/atom and for two H_2 molecules is $\Delta H = +0.056$ eV/atom.

To check the effect of cell relaxation on the electronic and dynamical properties, we report in Fig. 10 the electronic band structures, the dynamical and Eliashberg function of the theoretical P^{DFT}=0 kbar system with 2 H_2 molecules. As already mentioned, the band structure is practically unaffected, showing a metallic, low dispersive band at the Fermi level. The phonon band structure shows a overall dynamically stable phase which

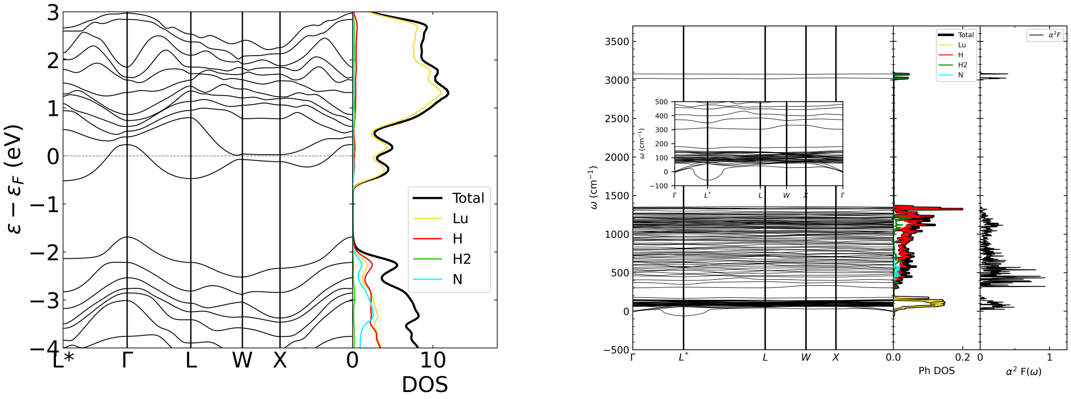

Supplementary Figure 10. Electronic dispersions and density of states (left) and dynamical properties and Eliashberg Function (right) of the $LuH_{2.875}N_{0.125}$ with two molecules structural relaxed up to theoretical P^{DFT}=0 kbar pressure.

underlines the crucial role the molecules play in the stabilization of the system. For example, if we consider the proposed Fm-3m phase, without molecules, the system shows a sever dynamical instability with large imaginary ($i400$ cm^{-1}) phonons extending over the whole Brillouin zone. While, the inclusion of molecules make most of the phonons dynamically stable with the only exception of a small instability limited around the L* point of the Brillouin Zone ($\omega(L^*) \sim i60$ cm^{-1}). This is a somehow expected result: a long-range distortion is indeed expected for a model-system which has a smaller unit cell than the one used for MD simulations, where we found the spontaneous formation of molecules, signaling the presence of (at least) orientational disorder of the hydrogen molecules in the system.

Finally, we also highlight that the phonon-DOS and, more importantly, the α^2F(ω) curve are practically indistinguishable from those obtained using experimental parameters, presented in the main text. The estimation of T_c for the relaxed P^{DFT}=0 kbar system (arbitrarily neglecting the contribution of the imaginary modes) is also in line with the estimation reported in the article (with T_c ∼20 K).

Supplementary Note 6. Electronic correlations treatment

In this section, we examine the potential need for a more detailed description of electron correlations, beyond the standard generalized gradient approximation, through a DFT+U approach. In literature, theoretical works suggest the use of Hubbard corrections for rare-

earth elements[2]: In particular, for Lu atoms, a Hubbard contribution of 5.5 eV was proposed for the f-orbitals, fitting this value to mimic the results obtained using hybrid functionals (HSE06) on LuN system, without hydrogens. The f-orbitals are deep in energy ($\sim -6 \div -8$ eV below the Fermi level), therefore the f-states do not take part in the chemical bonds and in the physical properties of the system we are interested in. Thus, as routinely done in studies on such systems, we can safely exclude the f-orbitals from the valence states in the pseudopotential. To validate this aspect, in Fig. 11 we report the density of states (DOS) obtained for $LuH_{2.875}N_{0.125}$ with two H_2 molecules with and without the inclusion of f-orbitals. Furthermore, when the f-states are included, we have highlighted the role of correlations including, or not, the Hubbard correction U= 5.5 eV. It is evident as the main features of the DOS around the Fermi level are unaffected by the presence of f-states neither by the Hubbard correction to that orbitals.

We thus move to consider the role of the eventual Hubbard correction for the d-states of Lu atoms. The Hubbard term proposed in literature for Lu-5d orbitals is U= 8.2 eV[2]. Also in this case, this large correction was determined through a fitting procedure with the aim to fit the hybrid functional results in a DFT+U framework, moreover for a system substantially different from ours (absence of hydrogen). Thus, we decided to rigorously re-calculate the Hubbard term for the Lu5d-states thought the method proposed by Cococcioni[3], as implemented in VASP. By using this scheme, we calculated the U correction for the different Lu atoms involved in our systems: near and far nitrogen.

The results are presented in Fig. 12: for Lu atoms bonded to N atoms we obtain a Hubbard correction $U_{5d} = 1.60$ eV and a $U_{5d} = 1.95$ eV for Lu atoms situated farther away from N (bonded with H). Accounting for the new value of the Hubbard term, we then highlighted this contribution on the electronic properties of our system. In Fig. 13, we report the density of states with and without the inclusion of Hubbard terms correction on the d-states.

The comparison between the DOS at the Fermi level clearly shows that the region around the Fermi level is, even in this case, qualitatively unaffected, meaning that the Hubbard correction does not alter the results and conclusions of our study. This is confirmed also by the calculation of the total energy difference between our new proposed molecular phase and the previously considered $LuN_{0.125}H_{2.875}$ phase, which results in line with what already calculated without inclusion of Hubbard correction on f- and d-states.

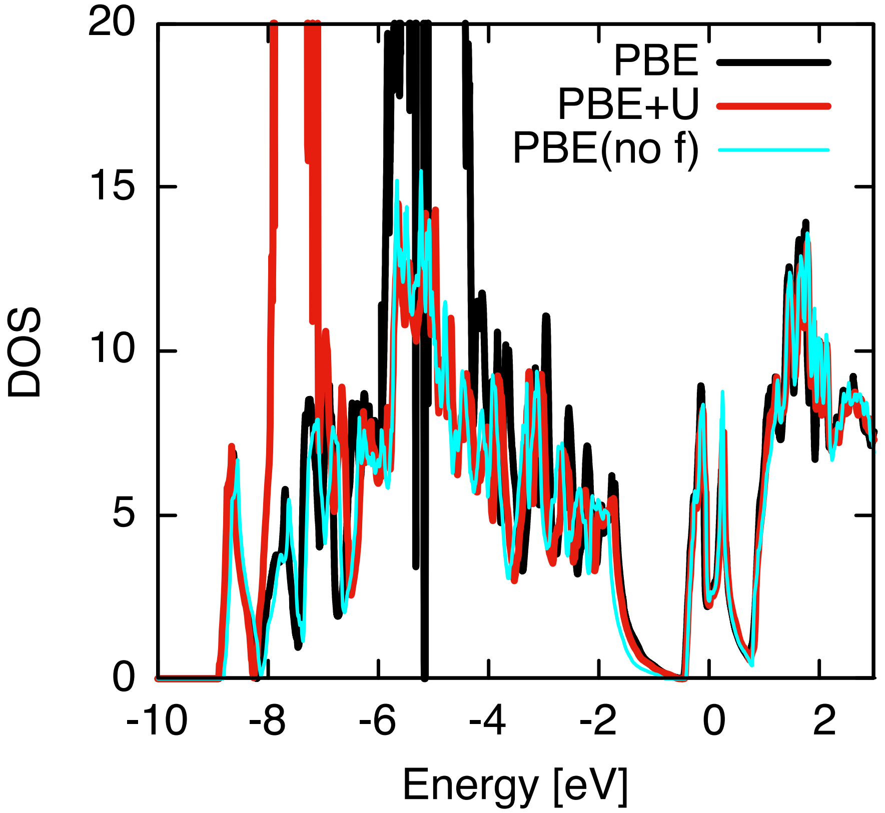

Supplementary Figure 11. Comparison between the densities of state for the $LuH_{2.875}N_{0.125}$ system with two H_2 molecules with and without the Hubbard correction on f states (U_f = 5.5 eV) in black and red respectively. For comparison we report also the DOS obtained without the inclusion of f-states in valence (cyan).

Additionally, structural and electronic parameters of the molecular hydrogen are robust with respect to the computational setup, as shown in Table II. The Bader charge analysis shows practically no change in the excess charge populating the H_2 orbitals, which results in a constant H-H bond length. Thus the over-delocalization of electrons of Lu atoms due to correlation errors does not impact the properties of molecular hydrogen to a sizable extent.

We can safely conclude that our computational approach correctly describes the chemical and physical properties of the Lu-N-H compound, even against the inclusion of additional contributions to electronic correlations.

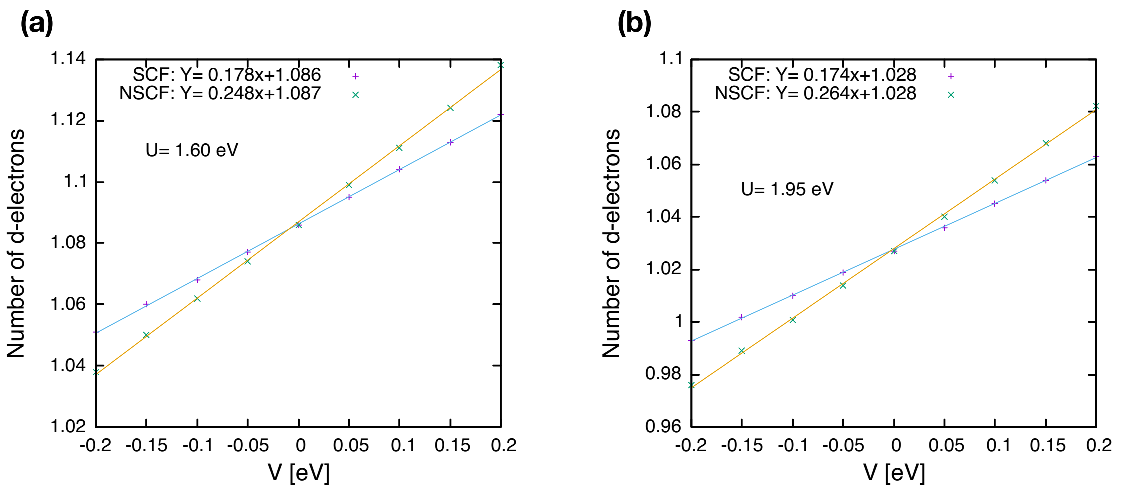

Supplementary Figure 12. In both graphs are represented the occupation number of *d*-orbitals as a function of the additional strength (in eV) of the spherical potential acting on the *d*-manifold for the self-consistent and the non self-consistent response, in the $LuH_{2.875}N_{0.125}$ system with two H_2 molecules. The values of the Hubbard term are computed for the Lu atom near N (panel a), with U= 1.6 eV, and far from N (panel b), with U= 1.95 eV.

U (eV)	Bader charge for H in H_2 (e)	H_2 bond length (Å)
0	1.08	0.84
1.6	1.08	0.84
4	1.10	0.83
6	1.11	0.83

Supplementary Table II. **Influence of the U parameters.** Bader charge of H in molecular form, H_2 bond length, and average lattice vectors as obtained for the N doped system (N in tetragonal site, and two H_2 molecules) by using different values of U correcting the electronic correlation on Lu-*d* orbitals.

Supplementary Note 7. More on the dynamical properties

In the following we will briefly discuss the dynamical properties of both the representative sub-systems described in Section Supplementary Note 5.

As shown in Fig.14, the representative sub-unit containing just one H_2 molecule results to

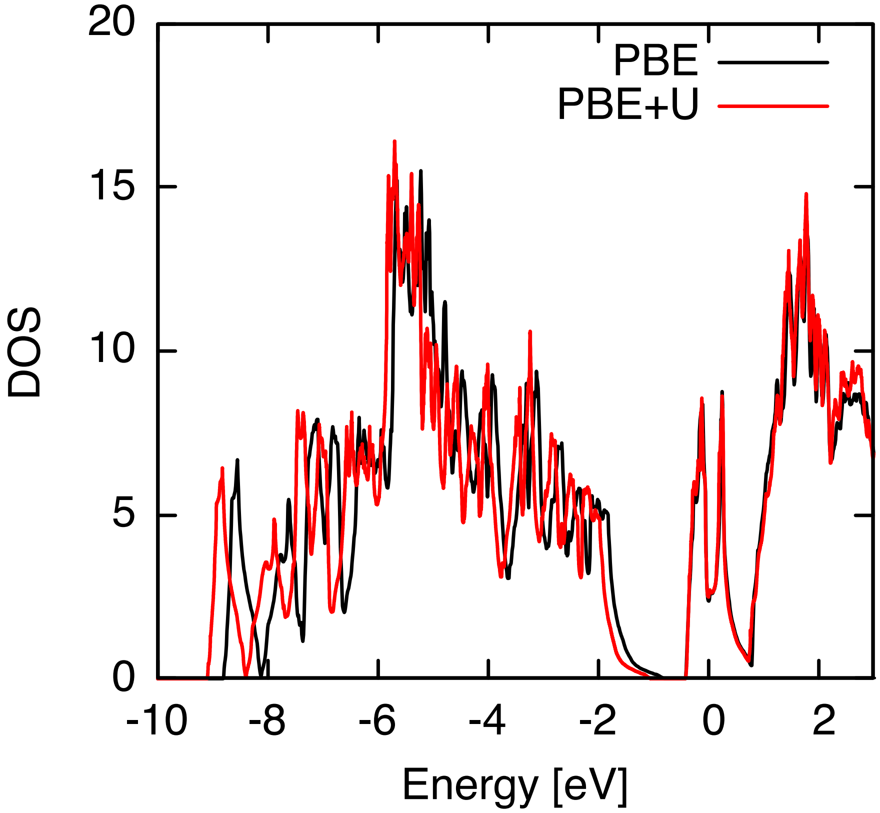

Supplementary Figure 13. Comparison between the DOS for the $LuH_{2.875}N_{0.125}$ system with two H_2 molecules with (red) and without (black) the Hubbard correction on d states ($U_d = 1.6$ eV).

be dynamically unstable at the harmonic level. The instability is not so deep and extended in the whole Brillouin zone, as in the case of the pristine $Fm\bar{3}m$ LuH_3 system, and is due to a widespread softening involving hydrogen atoms (see projections in Fig.14). The softening also affects other modes, at higher energies, still involving hydrogen atoms.

On the other hand, the representative sub-system containing two H_2 molecules result metallic (Fig.9) and dynamically stable.

Despite the instability observed for the first structure, the phonon dispersion and related DOS appear overall similar. In particular, the Lu-derived modes are found at low frequencies (<250 cm^{-1}), nearly separated from the other branches; N-related branches are located around 500 cm^{-1} and the H and H_2 modes extend up to ≃1500 cm^{-1}. The characteristic molecular vibrational modes are well separated from the rest of the spectrum at very high frequencies.

The subsystem, which consists of two H_2 molecules and exhibits dynamic stability, is ob-

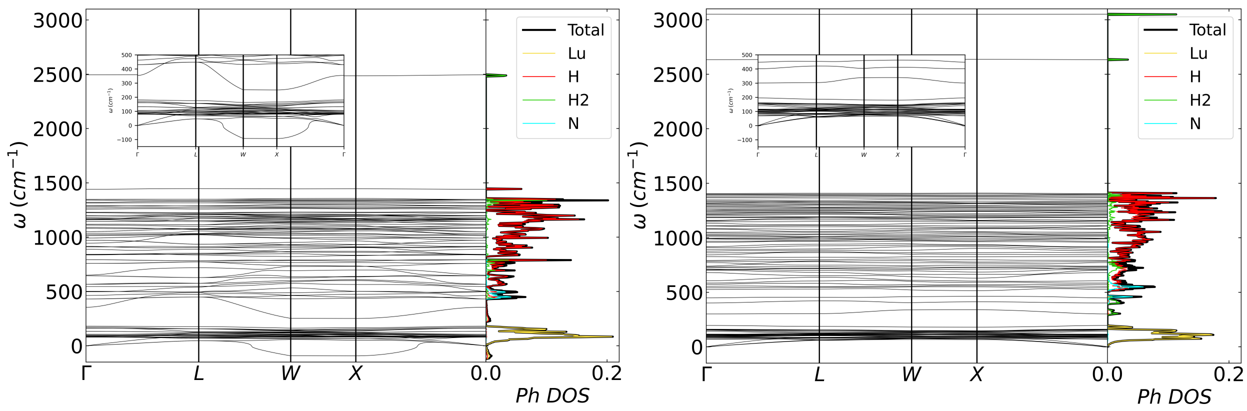

Supplementary Figure 14. Left panel: The phonon spectra obtained for $LuH_{2.875}N_{0.125}$ with one H_2 molecule. Inset: focus on low frequencies region showing the Lu modes (below 200 cm^{-1}) and the first softened modes around 250 cm^{-1}. Right panel: $LuH_{2.875}N_{0.125}$ in its metastable phase (2 molecules) phonon spectra. Inset: focus on low frequencies region showing the Lu modes (below 250 cm^{-1}) and the molecular *librational* modes (around $300 - 500$ cm^{-1}). Each plot is accompanied by its density of states. The hydrogen character of eigenvalues is highlighted (red the total H character, in green the molecular contribution, in cyan the nitrogen one, and in yellow the total Lu character).

served to be in a superconducting phase, featuring a total electron-phonon coupling strength of $\lambda = 0.66$.

As shown in Fig. 3 of the main paper, the function $\lambda(\omega) = 2\int_0^\omega \alpha^2 F(\omega')/\omega' d\omega'$ is characterized by two major steps: one at the low-energy (0 $cm^{-1} \leq \omega \leq 250$ cm^{-1}), due to Lu-modes, and the other starting from $\simeq 500$ cm^{-1} up to $\simeq 800$ cm^{-1} due to molecular and Nitrogen phonon modes (as evident from comparison with the projected phonon density of states). These last modes contribute more than 25% to the total λ.

To highlight the active role of H_2 molecules to the electron-phonon coupling, we have calculated the deformation potential for representative phonon modes in the region where $\lambda(\omega)$ is enhanced (at $\simeq 450$ cm^{-1}, $\simeq 550$ cm^{-1} and $\simeq 750$ cm^{-1}), highlighting the induced deformation of the electronic bands at the Fermi energy.

Results are presented in Fig.15(d),(e),(f).

The first mode, represented in Fig.15(a) mainly involve N, H_2 and H displacement and strongly couples with the electronic bands crossing the Fermi level, in particular for the states around the Γ point. The second mode we analyze, shown in Fig. 15(b), does not have

components on the H_2 molecule and indeed exhibits the lowest coupling with the electronic states at the Fermi energy (among the three modes here considered). On the other hand, the last mode (reported in Fig. 15(c)) moves only molecular and atomic hydrogen (without N) showing the largest coupling: strongly deforming the electronic bands at the Fermi energy. This analysis highlights how molecules strongly contribute to increase the electron-phonon coupling in the region $\simeq$ 450 - 750 cm^{-1} due to the strong deformation potential at the Fermi energy, with consequent impact on the superconducting phase.

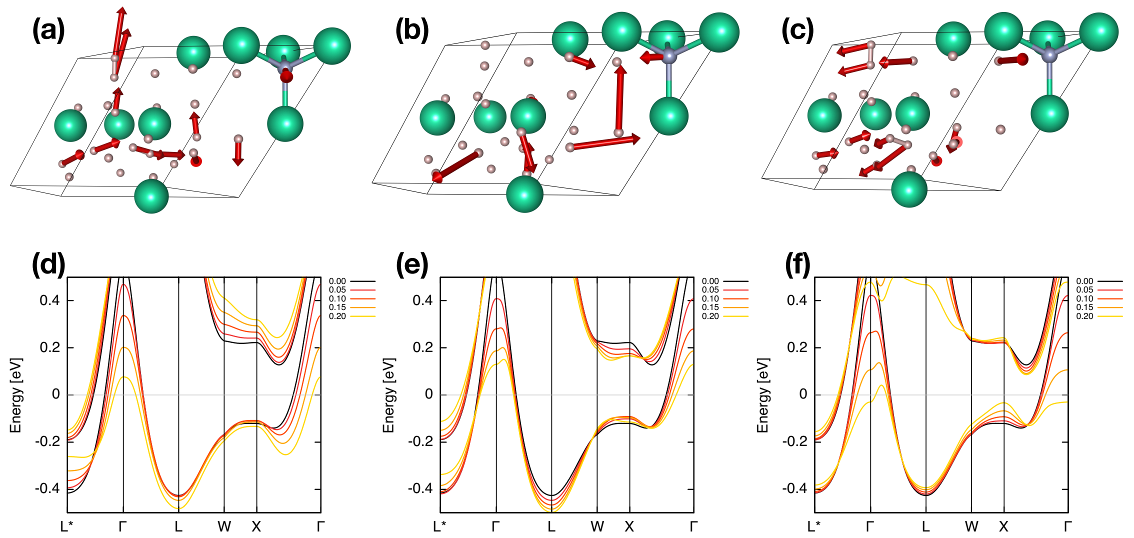

Supplementary Figure 15. The top row illustrates crystal structures along with eigenvectors corresponding to mode 27 at $\simeq$ 450 cm^{-1} (a), 29 at $\simeq$ 550 cm^{-1} (b) and 40 at $\simeq$ 750 cm^{-1} (c). To enhance clarity, only the eigenvectors making significant contributions are depicted in these figures. In the bottom row are shown the deformations of the bands at the Fermi energy for mode 27 (d), mode 29 (e) and mode 40 (f). In black is represented the pristine band structure for $LuH_{0.875}N_{0.125}$ while the color gradient show the deformation moving along the eigenvectors with different percetage on their modulus.

To understand the charge transfer accompanying the phonon modes and, thus, the relative role in determining the electron phonon coupling, we now analyze the Bader charge analysis as a function of the atomic displacements along all the 3 modes already considered.

As shown in Fig. 16, activating the atomic oscillation (we considered only positive displacement), we find a clear charge transfer mainly involving H-, H_2 and Lu atoms, which

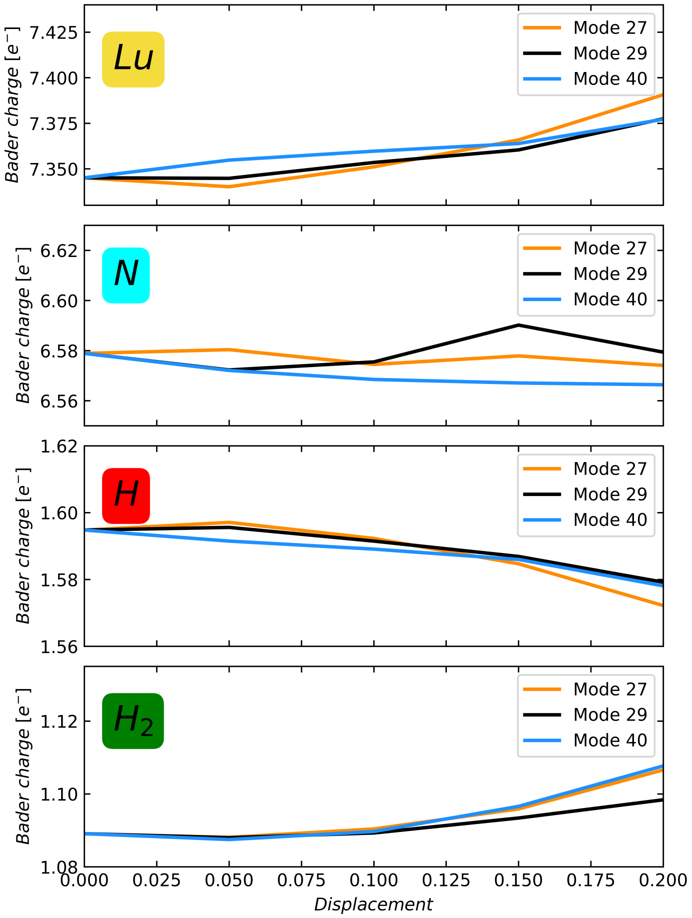

Supplementary Figure 16. We report the Bader charge analysis as obtained from VASP calculations as a function of the atomic displacements along the phonon eigenvectors we are interested in. The average value of the Bader charge is reported for every element (in units of the electronic charge, e).

show an appreciable relative variation (∼-1.9%, ∼1.8% and ∼ 0.7%, respectively). In particular, for the analyzed displacements, H- acts as donor, while H_2 and Lu as acceptors. In particular, the most coupled modes among the three analysed (27 and 40) give larger (and similar) charge transfer to the H_2; instead the less coupled mode (29) produces a smaller variation in the Bader charge of molecules. This aspect can be viewed as the real-space manifestation of the role of H_2 in determining the electron-phonon coupling of the representative

modes 27 and 40.

Supplementary Note 8. Calculation of the superconducting critical temperature

We used the $ab-initio$ Superconducting density functional theory (SCDFT)[4–6], an extension of DFT to the superconducting phase, to predict the superconducting critical temperature of the studied systems.

We solved the SCDFT gap equation using the functional form proposed in Ref. 7, in the isotropic approximation using the calculated α^2F and the Sham-Kohn approximation[8] for the electron self-energy for the normal state repulsion. The result are reported in Fig.17.

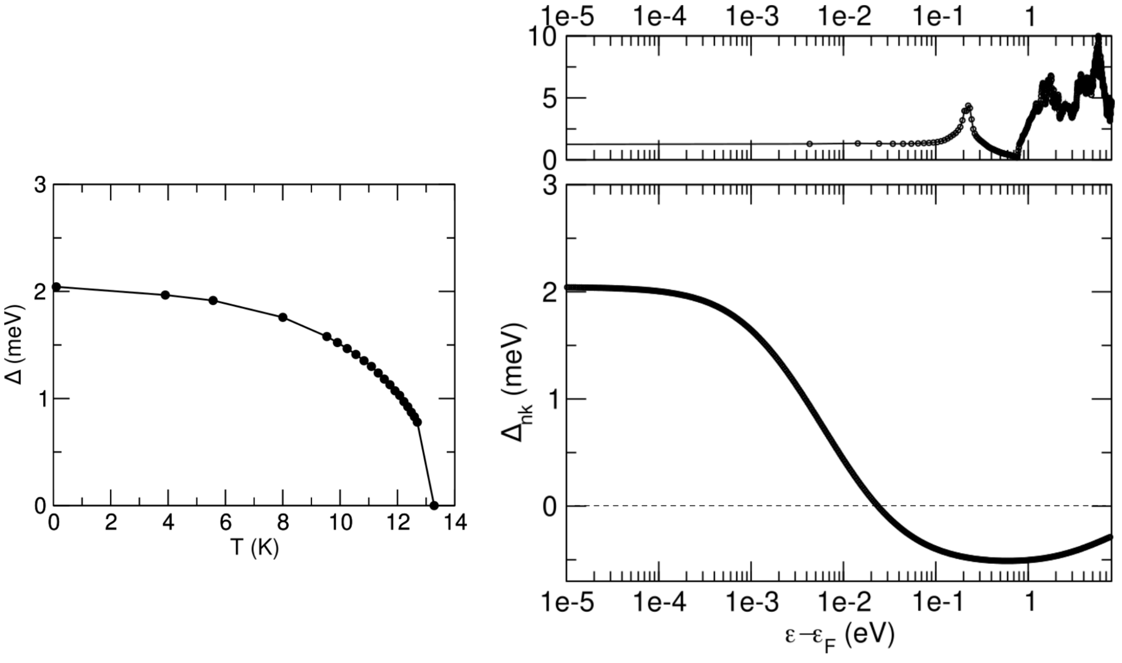

Supplementary Figure 17. Left panel: The superconducting energy gap as a function of temperature for the metallic phase with two H_2 molecules. Right panel: The superconducting energy gap at "zero" temperature as a function of the energy from the Fermi level and the electronic DOS in (states/spin/eV) on the same energy scale (on top).

The calculated superconducting gap at zero temperature is $\Delta(0) \simeq 2$ meV and it goes to zero at a critical temperature of $T_C \simeq 13$ K, with a ratio $\Delta(0)/K_BT_C$=1.78, in line with the BCS universal ratio of 1.764 for weak coupling superconductors.

The density of states close to the Fermi energy is nearly constant and the superconducting

gap results positive up to $\sim 10^{-2}$ meV, an energy distance of the order of the phononic energy range ($\omega_{log} \simeq 24$meV), then it changes sign at higher energies. Interestingly, the van Hove singularity falls into the negative region of the gap, contributing to the gap renormalization.

We have estimated the superconducting critical temperature also using Allen and Dynes formula resulting in $T_C \simeq 8$ K, with $\mu^* = 0.1$, slightly lower than the SCDFT value probably due to the different treatment of renormalization effects due to the Coulomb interaction.

Supplementary Note 9. Accuracy of MLFF-MD calculations

During the molecular MLFF-MD dynamics simulations, at each time step, the machine-learned force field predicts energy, forces, and the corresponding Bayesian error estimations [9, 10]. In our setup, if the Bayesian error is above a certain threshold, an *ab-initio* calculation is performed, and the reference energy and forces are added to the training data set. If the error is below the threshold, the ab-initio step is omitted, and the system is propagated via machine learning force-field predictions. The threshold is dynamically updated. Figure 18 shows the Bayesian error estimation on the forces (BEEF) as a function of time, and the corresponding threshold for the DFT calculations.

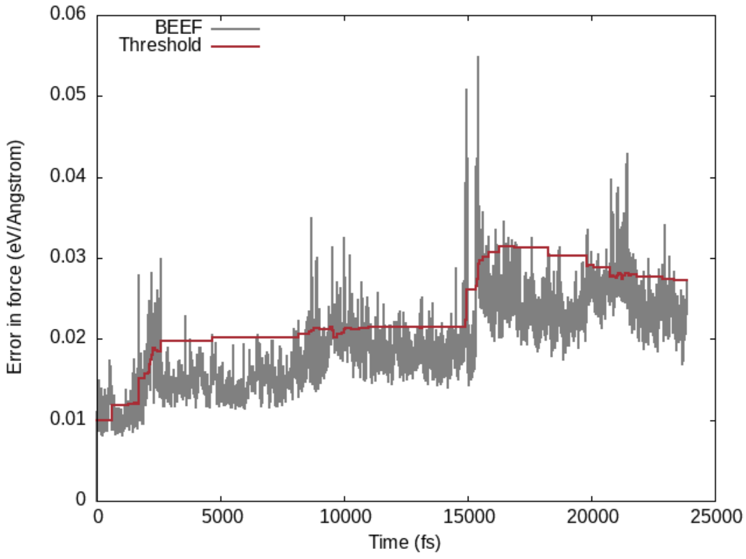

Supplementary Figure 18. Estimated errors in the MLFF-MD run at 300 K. The red line represents the threshold activating the DFT calculations; the gray line represents the Bayesian error estimate of forces (BEEF).

Supplementary Note 10. Comparison with XRD and Raman experimental spectra

In Fig.19 we report the comparison between the simulated XRD spectra obtained for a representative snapshot of the MD-trajectory with a recently reported experimental XRD measurement[11]. To calculate the XRD peaks we used the VESTA[12] code. The experimental points have been extracted by the spectra in Ref.[11]. Theory and experiments are in good agreement.

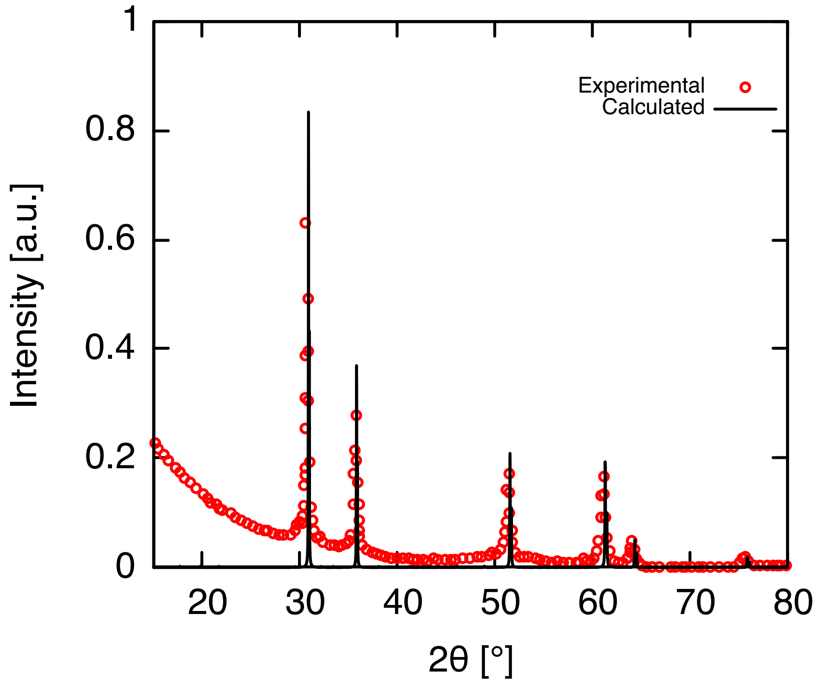

Supplementary Figure 19. Simulated XRD spectra (black line) obtained for a representative snapshot of the MD-trajectory compared with the experimental XRD from Ref.[11] (red circles).

In Fig.20 we report the comparison between the Raman spectra for the Lu-N-H ternary compound available in literature[1, 11, 13] with respect to the phononic density of states (without cross-section) relative to our "model" sub-systems (see Sec.Supplementary Note 5 on the SM). It is evident how the experimental Raman spectra are strongly dependent on the sample preparation, showing the appearance and/or disappearance of certain peaks depending on the thermodynamic treatment of the sample. It is useful to compare the theoretical frequencies obtained for both our "model" sub-systems (with $1H_2$ and $2H_2$ molecules

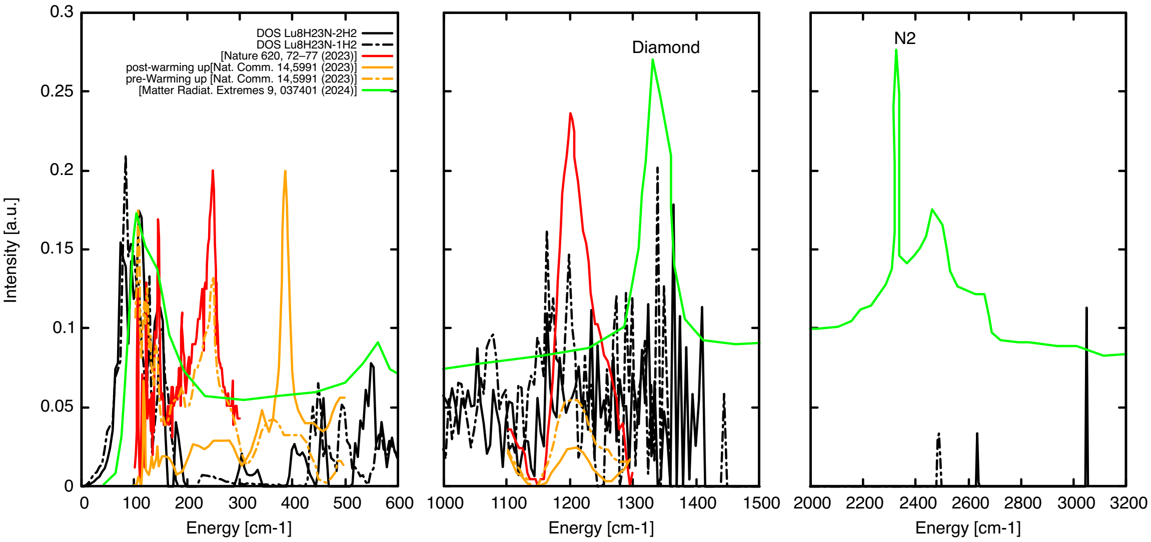

Supplementary Figure 20. Comparison between the experimental Raman spectra for the Lu-N-H ternary compound (red[11], orange[13] and green[1]) and the theoretical phonon density of states for our "model" sub-systems (black continuous ($2H_2$) and dashed lines ($1H_2$)).

per cell, respectively).

Unfortunately, to date, the only high frequency Raman data are those of the Lu-N-H ternary system in the (nominal) trigonal phase[1], we call for future experiments in the Lu-fcc crystal. Anyway, we can compare these results with our predicted spectra (in the Lu-fcc structure) considering that the trigonal structure is a deformation of the fcc-cubic lattice[14] and the very-high frequency region (above 2000 cm^{-1}) should be dominated by molecular modes.

The frequency range of the experimental Raman spectra are in overall agreement with our theoretical predictions.

[1] D. Wang, N. Wang, C. Zhang, C. Xia, W. Guo, X. Yin, K. Bu, T. Nakagawa, J. Zhang, F. Gorelli, P. Dalladay-Simpson, T. Meier, X. Lü, L. Sun, J. Cheng, Q. Zeng, Y. Ding, and H.-k. Mao, Unveiling a novel metal-to-metal transition in LuH2: Critically challenging superconductivity claims in lutetium hydrides, Matter and Radiation at Extremes **9**, 037401 (2024).

[2] M. Topsakal and R. Wentzcovitch, Accurate projected augmented wave (paw) datasets for rare-earth elements (re=la–lu), Computational Materials Science **95**, 263 (2014).

[3] M. Cococcioni and S. de Gironcoli, Linear response approach to the calculation of the effective interaction parameters in the LDA + U method, Phys. Rev. B **71**, 035105 (2005).

[4] L. N. Oliveira, E. K. U. Gross, and W. Kohn, Density-functional theory for superconductors, Phys. Rev. Lett. **60**, 2430 (1988).

[5] M. Lüders, M. A. L. Marques, N. N. Lathiotakis, A. Floris, G. Profeta, L. Fast, A. Continenza, S. Massidda, and E. K. U. Gross, Ab initio theory of superconductivity. i. density functional formalism and approximate functionals, Phys. Rev. B **72**, 024545 (2005).

[6] M. A. L. Marques, M. Lüders, N. N. Lathiotakis, G. Profeta, A. Floris, L. Fast, A. Continenza, E. K. U. Gross, and S. Massidda, Ab initio theory of superconductivity. ii. application to elemental metals, Phys. Rev. B **72**, 024546 (2005).

[7] A. Sanna, C. Pellegrini, and E. K. U. Gross, Combining eliashberg theory with density functional theory for the accurate prediction of superconducting transition temperatures and gap functions, Phys. Rev. Lett. **125**, 057001 (2020).

[8] L. J. Sham and W. Kohn, One-particle properties of an inhomogeneous interacting electron gas, Phys. Rev. **145**, 561 (1966).

[9] R. Jinnouchi, J. Lahnsteiner, F. Karsai, G. Kresse, and M. Bokdam, Phase transitions of hybrid perovskites simulated by machine-learning force fields trained on the fly with bayesian inference, Physical Review Letters **122**, 225701 (2019).

[10] R. Jinnouchi, F. Karsai, and G. Kresse, On-the-fly machine learning force field generation: Application to melting points, Physical Review B **100**, 014105 (2019).

[11] X. Ming, Y.-J. Zhang, X. Zhu, Q. Li, C. He, Y. Liu, T. Huang, G. Liu, B. Zheng, H. Yang, J. Sun, X. Xi, and H.-H. Wen, Absence of near-ambient superconductivity in $LuH_{2\pm x}N_y$,

Nature 10.1038/s41586-023-06162-w (2023).

[12] K. Momma and F. Izumi, VESTA 3 for three-dimensional visualization of crystal, volumetric and morphology data, Journal of Applied Crystallography **44**, 1272 (2011), arXiv:arXiv:1011.1669v3.

[13] X. Xing, C. Wang, L. Yu, J. Xu, C. Zhang, M. Zhang, S. Huang, X. Zhang, Y. Liu, B. Yang, X. Chen, Y. Zhang, J. Guo, Z. Shi, Y. Ma, C. Chen, and X. Liu, Observation of non-superconducting phase changes in nitrogen doped lutetium hydrides, Nature Communications **14**, 5991 (2023).

[14] B. Palosz, S. Stelmakh, E. Grzanka, S. Gierlotka, and W. Palosz, Application of the apparent lattice parameter to determination of the core-shell structure of nanocrystals, Zeitschrift für Kristallographie - Crystalline Materials **222**, 580–594 (2007).
